# Bee Venom Phospholipase A2 Ameliorates Atherosclerosis by Modulating Regulatory T Cells

**DOI:** 10.3390/toxins12100609

**Published:** 2020-09-23

**Authors:** Geun-Hyung Kang, Sujin Lee, Da Bin Choi, Dasom Shin, Jahee Kim, HyeJin Yang, Hyunsu Bae

**Affiliations:** Department of Physiology, College of Korean Medicine, Kyung Hee University, 26-6 Kyungheedae-ro, Dongdamoon-gu, Seoul 02453, Korea; geunhyung7@gmail.com (G.-H.K.); su11024@naver.com (S.L.); dphs0228@naver.com (D.B.C.); ssd060@naver.com (D.S.); rlawkgml1101@naver.com (J.K.)

**Keywords:** bee venom, phospholipase A2, PLA2, atherosclerosis, Tregs

## Abstract

Atherosclerosis is a chronic inflammatory disease caused by lipids and calcareous accumulations in the vascular wall due to an inflammatory reaction. Recent reports have demonstrated that regulatory T (Treg) cells have an important role as a new treatment for atherosclerosis. This study suggests that bee venom phospholipase A2 (bvPLA2) may be a potential therapeutic agent in atherosclerosis by inducing Treg cells. We examined the effects of bvPLA2 on atherosclerosis using *ApoE^-/-^* and *ApoE^-/-^*/Foxp3^DTR^ mice. In this study, bvPLA2 increased Treg cells, followed by a decrease in lipid accumulation in the aorta and aortic valve and the formation of foam cells. Importantly, the effect of bvPLA2 was found to depend on Treg cells. This study suggests that bvPLA2 can be a potential therapeutic agent for atherosclerosis.

## 1. Introduction

Atherosclerosis is a chronic inflammatory disease that causes cardiovascular heart disease. Lipid accumulation, a major cause of disease, occurs in the arterial walls and causes the narrowing of blood vessels by producing blood clots. The main risk factors of this disease include smoking, obesity, hypercholesterolemia, hypertension, and diabetes. It is well accepted that atherosclerosis is related to the innate and adaptive immune systems. In particular, T cells and macrophages are involved in the development of atherosclerosis. Atherosclerosis is ultimately caused by the interaction among endothelial cells, macrophages, smooth muscle cells, and lymphocytes, which are highly associated with lipid-laden macrophage foam cells and pro-inflammatory T cell factors [1,2,3,4].

It has been reported that secreted phospholipase A2 (sPLA2) is a disulfide-rich Ca^2+^-requiring enzyme that releases lysophospholipid products by catalyzing the sn-2 ester bond hydrolysis of glycerophospholipids. In addition, sPLA2 binds to a specific membrane receptor to act as a ligand to induce cellular signaling independent of enzymatic activity [5,6].

According to previous reports, group III PLA2 is involved in foam cell formation in macrophages. The formation of foam cells is triggered when monocyte-derived macrophages accumulate in the atherosclerotic lesion site or arterial wall where lipids are deposited. These monocyte-derived macrophages take up oxidized low-density lipoprotein (oxLDL) via a scavenger receptor. Moreover, foam cells cause an inflammatory response inside the arterial wall and are known to be an important component of atherosclerotic plaque formation [7].

CD4^+^ Foxp3^+^ regulatory T (Treg) cells are being investigated as an alternative method to reduce foam cells, and they are important factors in atherosclerosis [8]. In addition, they maintain immunologic homeostasis through the secretion of immunosuppressive cytokines and may have a potential therapeutic effect on atherosclerosis [9,10,11,12,13]. The importance of Treg cells has already been identified in several diseases.

Previously, we demonstrated the effect of bee venom phospholipase A2 (bvPLA2), a major active compound controlling the generation of Treg cells, in several immune-related diseases such as asthma, kidney disease, atopic dermatitis, and Alzheimer’s disease [14,15,16,17,18]. Many of the biological effects of PLA2s are dependent on their catalytic activity [19]. It also has been proposed that bvPLA2 increases the expression of prostaglandin E2 (PGE2), which then binds to the EP2 receptor in naïve T helper cells. Moreover, it induces differentiation into Treg cells [20].

Here, we hypothesized that bvPLA2 may reduce the risk of atherosclerosis caused by lipid accumulation and suppress the transformation of macrophages into foam cells by Treg cell. In this study, we confirmed a reduction of lipid accumulation and foam cell formation by treating with bvPLA2. Furthermore, we suggest that bvPLA2 may be a significant therapeutic agent to mitigate arteriosclerosis.

## 2. Results

### 2.1. Effect of bvPLA2 Treatment on Atherosclerotic Mice

**Figure 1:**
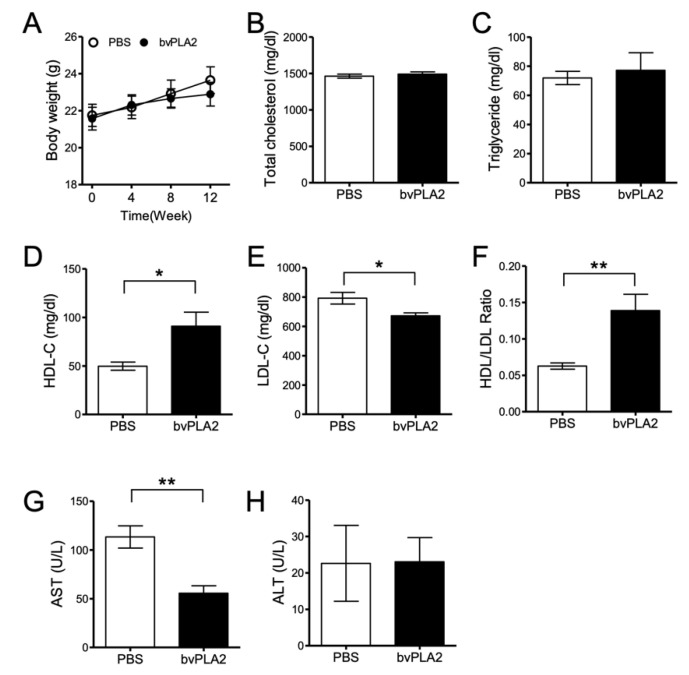
The effect of bee venom phospholipase A2 (bvPLA2) on atherosclerotic *ApoE^-/-^* mice. An atherogenic high-cholesterol diet (D12336) was fed to *ApoE^-/-^* mice for 12 weeks, and mice were then injected with PBS or bvPLA2 (0.2 mg/kg, i.p) every 2 days after a 4-week diet. The serum was separated from the blood and analyzed through a biochemical analyzer. (**A**) Body weight and diet were measured at 0, 4, 8, and 12 weeks. (**B**) Total cholesterol, (**C**) triglyceride, (**D**) high-density lipoprotein cholesterol (HDL-C), (**E**) low-density lipoprotein cholesterol (LDL-C), (**F**) HDL/LDL ratio, (**G**) aspartate aminotransferase (AST), and (**H**) alanine aminotransferase (ALT) were measured in the serum for lipid analysis. The data are displayed as the means ± SEM. The statistical analyses were conducted with Student’s unpaired *t* test; *, *p* < 0.05; **, *p* < 0.01 vs. the PBS group; n = 10.

**Figure A1:**
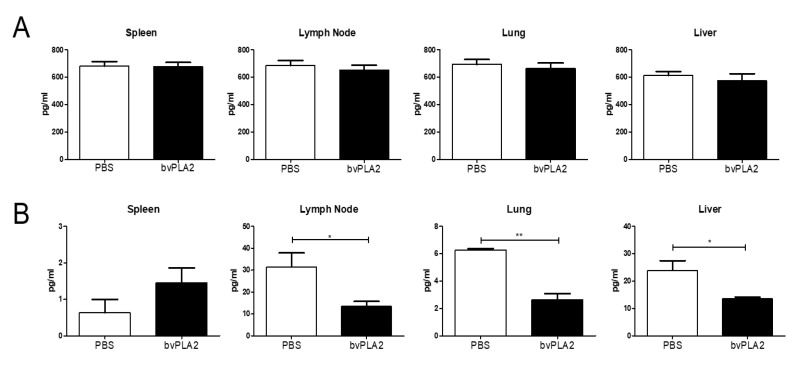
The effect of bvPLA2 on prostaglandin E2 (PGE2) and arachidonic acid (AA) levels in mice. Mice were injected with PBS or bvPLA2 (0.2 mg/kg, i.p) every 2 days for 5 times. After the injection, (**A**) PGE2 and (**B**) AA levels were analyzed in mouse spleen, lymph node, lung, and liver using ELISA system. The data are displayed as the means ± SEM. Statistical analyses were conducted with Student’s unpaired *t* test; *, *p* < 0.05; **, *p* < 0.01 vs. the PBS group; n = 3–4.

We investigated the therapeutic effect of bvPLA2 on atherosclerosis caused by a high-fat diet. Eight- to ten-week-old *ApoE* knock out (*ApoE^-/-^*) mice were used to induce hypercholesterolemia with an atherogenic high-cholesterol diet (D12336; Research Diets, Inc., New Brunswick, NJ, USA) for 12 weeks. The mice were divided into phosphate buffered saline (PBS) and bvPLA2 (0.2 mg/kg) groups and injected intraperitoneally (i.p.) every 2 days after a 4-week diet. Body weight was measured every week, and it increased less in the bvPLA2 group compared to that in the PBS group (Figure 1A). However, there was no significant difference between the two groups. Blood was also collected from the inferior vena cava to perform lipid analysis. Total cholesterol (TC) and triglyceride (TG) were not significantly different between those groups (Figure 1B,C). However, high-density lipoprotein cholesterol (HDL-C) was significantly increased (Figure 1D, *p* < 0.05, t = 2.739, df = 10) and low-density lipoprotein cholesterol (LDL-C) decreased (Figure 1E, *p* < 0.05, t = 2.694, df = 10) in the bvPLA2-treated group. The HDL/LDL ratio was significantly increased in the bvPLA2 group (Figure 1F, *p* < 0.01, t = 3.341, df = 10). Aspartate aminotransferase (AST) and alanine aminotransferase (ALT) were also measured to confirm the hepatotoxicity of bvPLA2. It was found that AST levels were significantly decreased in the bvPLA2 group (Figure 1G, *p* < 0.01, t = 4.214, df = 8), but there was no change in ALT (Figure 1H). To address the systemic effects of bvPLA2 on the production of bioactive lipids, the levels of prostaglandin E2 (PGE2) and arachidonic acid (AA) in vivo were analyzed. The results showed that bvPLA2 (0.2 mg/kg, i.p.) treatment did not induce the release of PGE2 or arachidonic acids in various tissues (Appendix A, Figure A1).

### 2.2. bvPLA2 Treatment Increases Regulatory T Cells in Lymph Nodes

**Figure 2:**
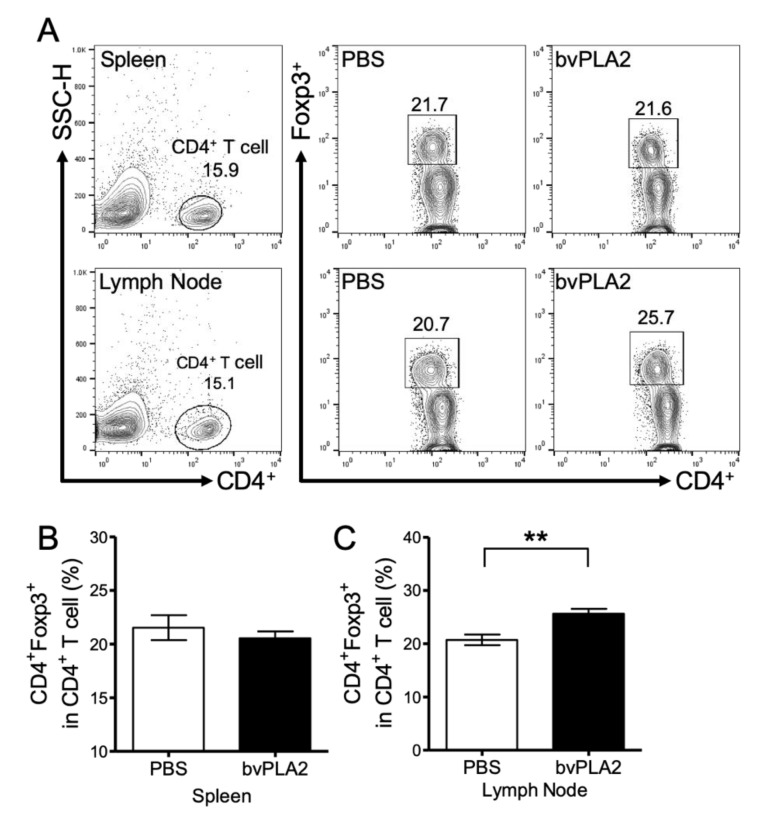
Effect of bvPLA2 on CD4^+^ Foxp3^+^ regulatory T cells. Single cells from the spleen and peripheral lymph node were measured using a fluorescence activated cell sorting (FACS) Calibur flow cytometer. (**A**) Representative flow cytometry plots from spleen (upper panel) and lymph node (lower panel) in *ApoE^-/-^* mice. Doublets and small multiplets were filtered by side-scatter height (SSC-H), and only CD4^+^ cells were gated. The cells were gated on Foxp3^+^ cells and analyzed. Number shows the percentage of CD4^+^ cells (left, upper and lower panel) out of total cells and Foxp3^+^ cells (right, upper and lower panel) in CD4^+^ cells with treatment of PBS and bvPLA2. Graphs show the percentage of CD4^+^ Foxp3^+^ Treg cells in CD4^+^ T cells in the spleen (**B**) and lymph node (**C**). The data are displayed as the means ± SEM. Statistical analyses were conducted with Student’s unpaired *t* test; **, *p* < 0.01 vs. the PBS group; n = 11.

To measure the increase of Treg cells following bvPLA2 treatment in the tissues, mouse spleens and peripheral lymph nodes (brachial, axial) were analyzed. Monocytes were isolated from the spleen and lymph nodes and analyzed by flow cytometry (Figure 2A). In the spleen, there was no difference in the CD4^+^ Foxp3^+^ Treg cell populations between the PBS and bvPLA2 groups (Figure 2B). On the other hand, there was a significant increase in CD4^+^ Foxp3^+^ Treg cells in the lymph nodes of the bvPLA2 group (Figure 2C, *p* < 0.01, t = 3.601, df = 20). This result is in accordance with previous results that the Treg cell populations quantitatively differ between the peripheral lymph nodes and the spleen to maintain homeostasis [21].

### 2.3. bvPLA2 Treatment Reduces the Inflammatory Cytokines

**Figure 3:**
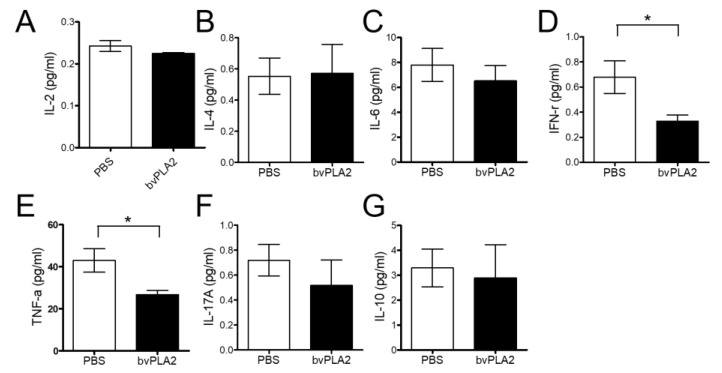
Effect of bvPLA2 on inflammatory cytokines. Inflammatory cytokines were analyzed and quantified in the serum by FACS analysis. The serum that separated from the blood was analyzed. The inflammatory cytokines, (**A**) IL-2, (**B**) IL-4, (**C**) IL-6, (**D**) IFN-γ, (**E**) TNF-α, (**F**) IL-17A, and (**G**) IL-10, were investigated. The data are displayed as the means ± SEM. The statistical analyses were conducted with Student’s unpaired *t* test; *, *p* < 0.05 vs. the PBS group; n = 9.

We analyzed and compared the inflammatory cytokines between PBS and bvPLA2 serum with a Cytometric Bead Array (CBA) Mouse Th1/Th2/Th17 Cytokine Kit. Interferon-gamma (IFN-γ) (Figure 3D, *p* < 0.01, t = 1.943, df = 14) and tumor necrosis factor-alpha (TNF-α) (Figure 3E, *p* < 0.01, t = 2.728, df = 16) serum levels significantly decreased in the bvPLA2 group compared to those in the PBS group. However, there was no significant difference in interleukin (IL)-2, IL-4, IL-6, IL-17A, and IL-10 (Figure 3A–C,F,G). This result suggests that bvPLA2 may potentially play a role in alleviating atherosclerosis by reducing cytokines.

### 2.4. bvPLA2 Treatment Reduces Atherosclerotic Lesions and Foam Cell Formation

**Figure 4:**
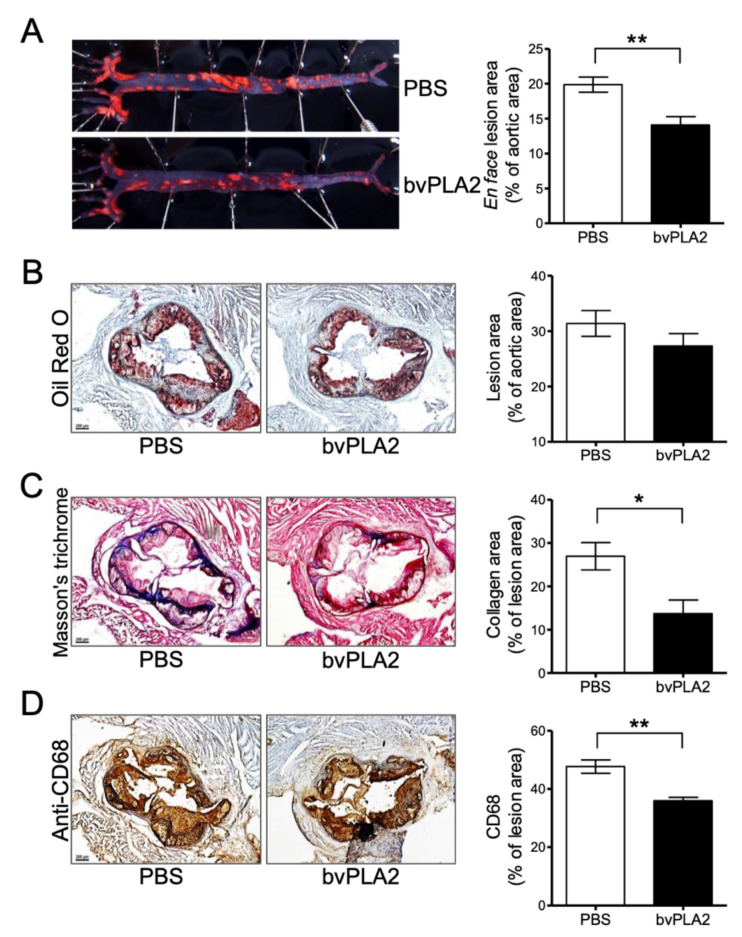
The therapeutic effect of bvPLA2 on atherosclerosis. (**A**) *ApoE^-/-^* mice aortas were measured with Oil red O staining and confirmed with an “en face” method. (**B**) Lipid accumulation of the aortic valve with Oil red O, (**C**) fibrosis with Masson’s trichrome (MT), (**D**) foam cell formation by macrophage with immunohistochemistry (IHC) staining were measured and quantified in heart sections (magnification ×50). The data are displayed as the means ± SEM. Statistical analyses were conducted with Student’s unpaired *t* test; *, *p* < 0.05; **, *p* < 0.01 vs. the PBS group; n = 6.

We confirmed the effect of Treg cells induced by bvPLA2 on atherosclerosis via the “en face” method. We extracted the aorta of mice fed an atherogenic diet for 12 weeks and measured the aorta with Oil red O staining. Indeed, the incidence of atherosclerosis was dramatically reduced in the bvPLA2-treated group (14.11% ± 1.19%) compared to that in the PBS group (19.87% ± 1.08%) (Figure 4A, *p* < 0.01, t = 2.967, df = 10). Furthermore, the accumulation of lipid on the aortic valve was decreased, although this was not significant (Figure 4B). Collagen staining with Masson’s trichrome (MT) was performed to confirm fibrosis in the heart. There was a significant reduction in fibrosis in the bvPLA2 group compared to that in the PBS group (Figure 4C, *p* < 0.05, t = 1.943, df = 14). Then, we identified and quantified foam cells through immunohistochemistry (IHC) using anti-CD68, which is a macrophage marker. Macrophages that developed into foam cells were significantly decreased in the bvPLA2 group (Figure 4D, *p* < 0.01, t = 4.528, df = 8). In this experiment, we found that bvPLA2-induced Treg cells may have a therapeutic effect on atherosclerosis by inhibiting foam cell formation along with reducing fibrosis.

### 2.5. Effect of bvPLA2 Treatment on ApoE^-/-^/Foxp3^DTR^ Mice

**Figure 5:**
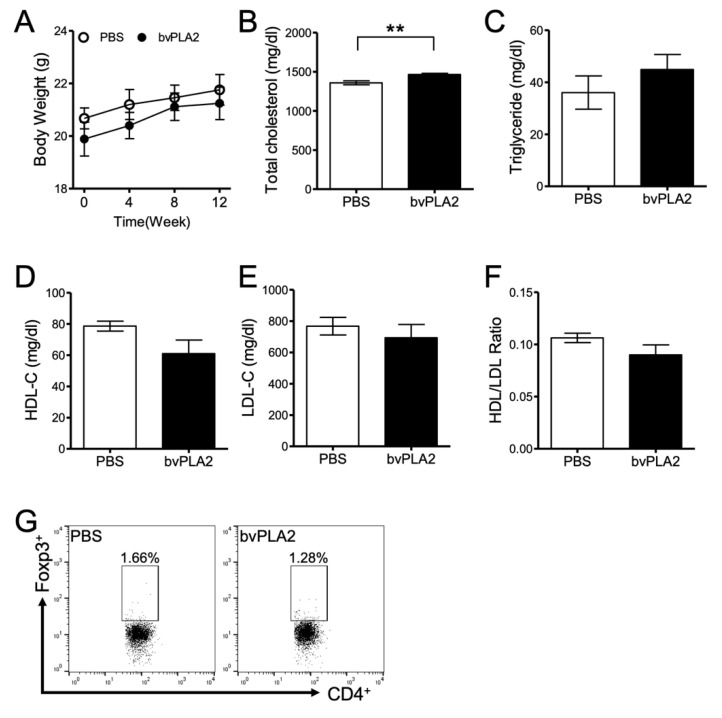
The effect of bvPLA2 on *ApoE^-/-^*/Fopx3^DTR^ mice. *ApoE^-/-^/*Fopx3^DTR^ mice were fed an atherogenic diet for 12 weeks, as in the previous experiment. They were injected with PBS or bvPLA2 (0.2 mg/kg, i.p) every 2 days after a 4-week diet. In addition, 1 µg of diphtheria toxin (DT) was injected for two consecutive days to deplete Tregs. (**A**) Body weight was measured weekly and shown at 0, 4, 8, and 12 weeks. (**B**) Total cholesterol, (**C**) triglyceride, (**D**) HDL-C, (**E**) LDL-C, and (**F**) HDL/LDL ratio were measured for lipid analysis in the serum. (**G**) Treg depletion was confirmed in the peripheral lymph node by FACS analysis. The cells were gated on CD4^+^ Foxp3^+^ cells and analyzed. Number showed the percentage of CD4^+^ Foxp3^+^ cells in total cells with treatment of PBS (**left panel**) and bvPLA2 (**right panel**). The data are displayed as the means ± SEM. The statistical analyses were conducted with Student’s unpaired *t* test; **, *p* < 0.01 vs. the PBS group; n = 7.

We used the *ApoE^-/-^/*Foxp3^DTR^ mice to confirm whether bvPLA2 has therapeutic effects on Treg depletion. *ApoE^-/-^/*Foxp3^DTR^ mice fed an atherogenic diet for 12 weeks were injected i.p. with either PBS or bvPLA2 (0.2 mg/kg) every 2 days after a 4-week diet. For Treg depletion, *ApoE^-/-^*/Foxp3^DTR^ mice were injected i.p. with 1 µg of diphtheria toxin (DT) for two consecutive days during the atherogenic diet. There were no significant differences in body weight, TG, HDL-C, LDL-C, or HDL/LDL ratio between the PBS and bvPLA2 groups (Figure 5A,C–F). However, the total cholesterol slightly increased in the bvPLA2 group (Figure 5B, *p* < 0.01, t = 3.464, df = 9). Treg depletion was confirmed by flow cytometry analysis using mouse peripheral lymph node cells (Figure 5G). Thus, we confirmed that bvPLA2 exerts its functions depending on the presence of Treg.

### 2.6. Effects of Treg Deletion on the Treatment of Arteriosclerosis with bvPLA2

**Figure 6:**
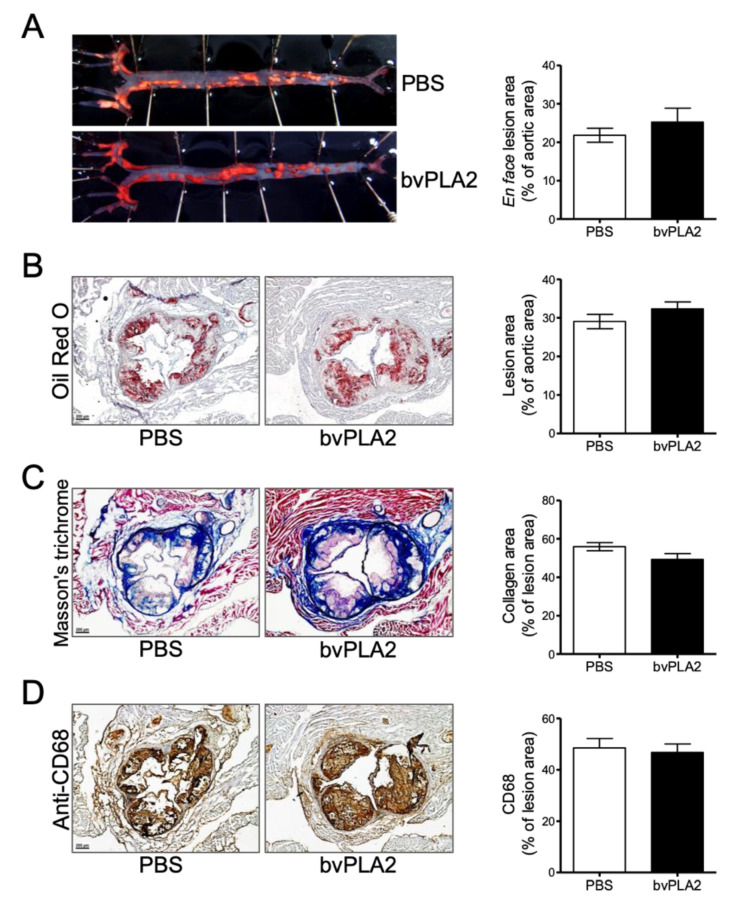
The therapeutic effect of bvPLA2 treatment on the atherosclerotic lesions of Treg-depleted mice. Atherosclerotic lesions were confirmed in the aorta of Treg-depleted mice. (**A**) The aorta was measured and quantified with Oil red O staining and an “en face” method. (**B**) Lipid accumulation of the aortic valve, (**C**) fibrosis, and (**D**) foam cell formation were measured and quantified in heart sections (magnification ×50). The data are displayed as the means ± SEM (n = 5). The statistical analyses were conducted with Student’s unpaired *t* test.

We also performed the same experimental “en face” method and Oil red O staining with the aorta from *ApoE^-/-^*/Foxp3^DTR^ mice. There were no significant differences in lipid accumulation in the Treg-depleted mouse aortas of the PBS and bvPLA2 groups (Figure 6A,B). Fibrosis was measured and quantified by MT staining, and foam cell formation was also confirmed with IHC staining. However, there were no significant differences (Figure 6C,D). These results suggest that the therapeutic effect of bvPLA2 is dependent on Treg cells in the atherosclerotic lesion.

## 3. Discussion

Atherosclerosis is a chronic inflammatory disease caused by lipid and calcareous accumulation. It leads to blood vessel narrowing and clogging and eventually death. The main cause of atherosclerosis is thought to be an inflammatory reaction by foam cells, in which monocyte-derived macrophages take up oxLDL. Current treatments of atherosclerosis include drugs of the statin family, angioplasty, or stent implantation. Recently, Treg cells were mentioned as an alternative therapy for atherosclerosis [10,12,13]. Foks et al. and Lin et al. mentioned that Treg cells are involved in foam cell formation, cholesterol metabolism, and immune suppression [11,22]. In previous studies, it was shown that increased Treg cells inhibited atherosclerosis [23]. Conversely, Treg cell deficiency further induced atherosclerosis [24]. Based on these studies, we examined the therapeutic effect of increased Treg cells by bvPLA2 on atherosclerosis.

Previously, several reports confirmed the effect of bvPLA2 according to the dosage administration and experimental systems. The activity of bvPLA2 is well studied by the experimental methods—especially, the adverse effect induced by bvPLA2 when it is injected directly into the paw and spinal cord at a wide range (ng–µg) of dosages [25,26,27], and on the other hand, the beneficial effect of bvPLA2 found when it is injected peritoneally at 0.1–1 mg/kg in an in vivo study [14,15,20,28,29]. Based on these results, we selected and applied 0.2 mg/kg of bvPLA2 to show the therapeutic effect in atherosclerosis.

Prior to confirming the effect of bvPLA2 on atherosclerosis, the biologically active lipid metabolites (PGE2 and AA) were analyzed to exclude the potential effects of bvPLA2 on the systemic production of PGE2 and AA. This demonstrated that PGE2 and AA levels were not increased by bvPLA2 treatments, suggesting that a relatively low dosage of bvPLA2 (0.2 mg/kg) did not affect lipid metabolite production systemically. These results implicated that the biological effects of bvPLA2 on Treg inductions could be mediated mainly by the paracrine production of biological lipids at sites where Treg cells were differentiated, such as at lymph nodes and the spleen.

We confirmed that bvPLA2 did not affect total cholesterol, but bvPLA2 increased HDL and decreased LDL and AST significantly (Figure 1). However, bvPLA2 treatment following Treg cell depletion, increased cholesterol but did not affect changes in LDL and HDL. From these results, it was confirmed that the increased Treg cells by bvPLA2 affected lipoprotein production, and its further anti-atherogenic effect was confirmed.

*ApoE^-/-^* and *ApoE^-/-^*/Foxp3^DTR^ mice were used to establish an atherosclerosis model by consuming a high-cholesterol diet according to several reports [30,31]. In this study, we found that bvPLA2 treatment increased Treg cells in the peripheral lymph nodes of *ApoE^-/-^* mice (Figure 2). Treg cells expanded by bvPLA2 also reduced several inflammatory cytokines (Figure 3). Moreover, these results had an effect on foam cell formation and lipid accumulation in the aorta (Figure 4). We examined whether the increased Treg cells by bvPLA2 were associated with suppressed foam cell formation and reduced inflammatory reactions. Interestingly, these results did not appear when Treg cells were depleted (Figure 5 and Figure 6). Upon Treg depletion, bvPLA2 only affected total cholesterol and HDL-C (Figure 5). The effect of bvPLA2 was also apparent in the atherosclerotic aorta (Figure 6). Based on these results, bvPLA2 has an anti-atherosclerotic effect that is dependent on Treg cells.

In this study, bvPLA2 increased HDL-C and decreased LDL-C by increasing Treg cells (Figure 1D–F). It is known that HDL-C, good cholesterol, plays an important role in cardiovascular-related disease. Further, LDL and HDL are important for determining the risk of coronary artery disease (CAD) [32]. Rueda et al. reported the relationship between HDL-C and Treg cells, revealing that HDL-C is involved in Treg cell survival and viability [33]. However, the mechanism of bvPLA2 on cholesterol remains to be established. Our results suggest that bvPLA2 has an important role in atherosclerosis by modulating Treg cells. These results may useful in developing new treatments for other heart diseases.

## 4. Material and Methods

### 4.1. Mice

Male 8- to 10-week-old *ApoE^-/-^* mice (B6.129P2-*Apoe*^tm1Unc^/J) and C57BL/6J mice were purchased from Jackson Laboratories (OrientBio, Seongnam, Korea). *ApoE^-/-^* mice were crossed with Foxp3^DTR^ mice (B6.1 29(Cg)-*Foxp3^tm3(DTR/GFP)Ayr^*/J) to generate *ApoE^-/-^*/Foxp3^DTR^ mice. *ApoE^-/-^* and *ApoE^-/-^*/Foxp3^DTR^ mice were fed an atherogenic high-cholesterol diet (D12336; Research Diets, Inc., New Brunswick, NJ, USA) containing 35% fat, 1.25% cholesterol, and 0.5% sodium cholate. This atherogenic diet enhances plasma cholesterol levels to double in hypomorphic ApoE mice, unlike the Western diet without cholate [34]. The mice were fed an atherogenic diet for 12 weeks, and PBS or bvPLA2 (0.2 mg/kg, Sigma-Aldrich, St. Louis, MO, USA) were injected intraperitoneally (i.p.) every 2 days after a 4-week diet. bvPLA2 was dissolved in PBS as a vehicle. For Treg depletion experiments, *ApoE^-/-^*/Foxp3^DTR^ male mice were given i.p. injections of 1 µg diphtheria toxin (DT, Sigma-Aldrich), dissolved in PBS, for two consecutive days. After 12 weeks of a high-fat diet, the animals were sacrificed for tissue sampling after 8 h of fasting. All mice were housed in conventional plastic cages with free access to feed and water at 23 ± 2 °C, 60% ± 10% humidity, and a 12-h light/dark photoperiod. The study was conducted following the Rules for Animal Care and the Guiding Principles for Animal Experiments Using Animals and was approved on 4. November 2016 by the University of Kyung Hee Animal Care and Use Committee (KHUASP(SE)-16-119).

### 4.2. Body Weight and Lipid Profile

The body weight and diet intake of mice were monitored every week. Blood samples were collected from the inferior vena cava and centrifuged (1000× *g* at 4 °C for 10 min) to separate the serum. Total cholesterol, triglyceride, high-density lipoprotein (HDL-C), low-density lipoprotein (LDL-C), AST, and ALT were determined using a Beckman Coulter AU480 automatic biochemistry analysis system with kit reagents provided by the manufacturer (Beckman Coulter, Inc., Brea, CA, USA).

### 4.3. Flow Cytometric Analysis

Flow cytometric analysis of regulatory T (Treg) cells was performed using phycoerythrin (PE)-conjugated anti-mouse CD4 (clone RM4-5; BD Biosciences, San Diego, CA, USA) and Alexa Fluor 647 anti-mouse/rat Foxp3 antibodies (clone MF23; BD Biosciences). Treg depletion was confirmed using allophycocyanin (APC)-conjugated anti-mouse CD4 antibody (clone 145-2C11; eBioscience, San Diego, CA, USA). Spleens and peripheral lymph nodes were isolated and separated into single cells using 25 µm nylon mesh (BD Biosciences). After treatment with red blood cell (RBC) lysis buffer (BD Biosciences), the cells were washed twice with PBS and stained with PE-conjugated anti-mouse CD4 antibody in staining buffer (BD Biosciences) on ice for 30 min in the dark. The cells were washed twice with PBS, and fixation/permeabilization buffer (eBioscience) was added for 15 min at room temperature (RT). Subsequently, the cells were washed twice with PBS and stained with Alexa Fluor 647 anti-Foxp3 antibody in the dark at 4 °C for 1 h. Then, the samples were analyzed with a FACS Calibur flow cytometer (BD Biosciences) and FLOWJO software (Tree Star, Ashland, OR, USA).

### 4.4. ELISA and Inflammatory Cytokines Analysis

Mouse prostaglandin E2 (PGE2, Enzo, Farmingdale, NY, USA) and arachidonic acid (AA, Aviva systems biology, San Diego, CA, USA) levels were measured by using enzyme-linked immunosorbent assay (ELISA) kits according to the manufacturer’s instructions. Inflammatory cytokine levels in serum were analyzed with a Cytometric Bead Array (CBA) Mouse Th1/Th2/Th17 Cytokine Kit (BD Biosciences) according to the protocol recommended by the manufacturer. Serum was obtained from blood samples by centrifuging 1000× *g* at 4 °C for 10 min. A mixture of captured antibody beads and PE-conjugated antibody was added to the serum and incubated at RT. After washing with PBS, the data were acquired using a BD FACS Calibur flow cytometer and analyzed using BD CellQuest, BD CBA software (BD Biosciences). Individual cytokine concentrations were indicated by their fluorescence intensities.

### 4.5. Measurement of Atherosclerotic Lesions in the Aorta

To measure the atherosclerotic lesion of the aorta, aortas were extracted and fixed with 10% formalin. After washing with PBS, an incision was performed using the “en Face” method. The iliac arteries were also incised and fixed. The fixed aortas were stained with Oil red O, and images of atherosclerotic lesions were obtained a camera (Samsung, Seoul, Korea). Total aortic surface area and Oil red O staining area were measured using Adobe Photoshop software version 5.0.1 (Adobe Systems, San Jose, CA, USA).

### 4.6. Histological Analysis

The heart and ascending aorta were embedded into optical cutting temperature (OCT) compound to measure lipid accumulation in the aortic sinus. Frozen tissues were sectioned into 7 µm thick sections. The sections that contained the cusps of 3 aortic valves at the junction of the aorta and left ventricle were selected. Oil red O staining was performed to confirm the lipid accumulation in the aortic sinus. Images were obtained using a Zeiss inverted microscope (Axio Observer 5), and the atherosclerotic lesions were observed. Lipid accumulation in the aortic sinus was measured using Adobe Photoshop software version 5.0.1 (Adobe Systems, San Jose, CA, USA). Masson’s trichrome (MT) staining was performed to measure fibrosis in the aortic sinus. Immunohistochemical (IHC) staining was also carried out to examine foam cell formation. Anti-CD68 antibody (clone H-255; Santa Cruz Biotechnology, Dallas, TX, USA) was used for IHC staining at a 1:50 concentration. MT staining and IHC staining were analyzed using ImageJ Fiji software (WS Rasband, National Institutes of Health, Bethesda, MD, USA). The quantitation was calculated as the percentage of the total aortic sinus versus the stained portion.

### 4.7. Statistical Analysis

Statistical analysis of the data was performed using Prism 7 software (GraphPad Software Inc., La Jolla, CA, USA). The results were expressed as the means ± SEM. Two-tailed Student’s unpaired test was performed to compare the two groups.
